# Once-Daily Dolutegravir–Based Antiretroviral Therapy Among Adults With Advanced HIV Disease Receiving 1 Month of Daily Rifapentine and Isoniazid for Tuberculosis-Preventive Therapy and Fluconazole for Treatment of Cryptococcal Meningitis: Results of a Pharmacokinetic Study

**DOI:** 10.1093/ofid/ofag415

**Published:** 2026-07-08

**Authors:** Jayne Ellis, Gila Hale, Allan Buzibye, Denis Omali, Sandra Naluyima, Nathan Ntenkaire, Laura J Nsangi, Jane Frances Ndyetukira, Alisat Sadiq, Cynthia Ahimbisibwe, Shifah Nabbaale, Asmus Tukundane, Wilber Bakka, Jane Gakuru, Aida N Kawuma, Fiona V Cresswell, David A J Moore, David B Meya, David R Boulware, Catriona Waitt, Joseph N Jarvis, Melanie R Nicol

**Affiliations:** Clinical Research Department, Faculty of Infectious and Tropical Diseases, London School of Hygiene and Tropical Medicine, London, United Kingdom; Infectious Diseases Institute, College of Health Sciences, Makerere University, Kampala, Uganda; Clinical Research Department, Faculty of Infectious and Tropical Diseases, London School of Hygiene and Tropical Medicine, London, United Kingdom; Infectious Diseases Institute, College of Health Sciences, Makerere University, Kampala, Uganda; Infectious Diseases Institute, College of Health Sciences, Makerere University, Kampala, Uganda; Infectious Diseases Institute, College of Health Sciences, Makerere University, Kampala, Uganda; Infectious Diseases Institute, College of Health Sciences, Makerere University, Kampala, Uganda; Infectious Diseases Institute, College of Health Sciences, Makerere University, Kampala, Uganda; Infectious Diseases Institute, College of Health Sciences, Makerere University, Kampala, Uganda; Infectious Diseases Institute, College of Health Sciences, Makerere University, Kampala, Uganda; Infectious Diseases Institute, College of Health Sciences, Makerere University, Kampala, Uganda; Infectious Diseases Institute, College of Health Sciences, Makerere University, Kampala, Uganda; Infectious Diseases Institute, College of Health Sciences, Makerere University, Kampala, Uganda; Infectious Diseases Institute, College of Health Sciences, Makerere University, Kampala, Uganda; Infectious Diseases Institute, College of Health Sciences, Makerere University, Kampala, Uganda; Infectious Diseases Institute, College of Health Sciences, Makerere University, Kampala, Uganda; Infectious Diseases Institute, College of Health Sciences, Makerere University, Kampala, Uganda; Clinical Research Department, Faculty of Infectious and Tropical Diseases, London School of Hygiene and Tropical Medicine, London, United Kingdom; Infectious Diseases Institute, College of Health Sciences, Makerere University, Kampala, Uganda; Global Health and Infection, Brighton and Sussex Medical School, Brighton, United Kingdom; Clinical Research Department, Faculty of Infectious and Tropical Diseases, London School of Hygiene and Tropical Medicine, London, United Kingdom; Infectious Diseases Institute, College of Health Sciences, Makerere University, Kampala, Uganda; Division of Infectious Diseases and International Medicine, Department of Medicine, University of Minnesota, Minneapolis, Minnesota, USA; School of Medicine, College of Health Sciences, Makerere University, Kampala, Uganda; Division of Infectious Diseases and International Medicine, Department of Medicine, University of Minnesota, Minneapolis, Minnesota, USA; Infectious Diseases Institute, College of Health Sciences, Makerere University, Kampala, Uganda; Department of Women’s and Children’s Health, Faculty of Health and Life Sciences, University of Liverpool, Liverpool, United Kingdom; Clinical Research Department, Faculty of Infectious and Tropical Diseases, London School of Hygiene and Tropical Medicine, London, United Kingdom; Botswana Harvard Health Partnership, Gaborone, Botswana; Department of Experimental and Clinical Pharmacology, College of Pharmacy, University of Minnesota, Minneapolis, Minnesota, USA

**Keywords:** dolutegravir, fluconazole, rifapentine, pharmacokinetics, tuberculosis-preventive therapy

## Abstract

**Background:**

One month of daily isoniazid and rifapentine (1HP) is an efficacious, well-tolerated, and safe tuberculosis-preventive therapy regimen. Its implementation has been slow due to concern about drug-drug interactions with rifapentine.

**Methods:**

This single-arm pharmacokinetic study included 15 adults with advanced human immunodeficiency virus (HIV) disease receiving once-daily 50-mg dolutegravir-based antiretroviral therapy during 1HP therapy, alongside once-daily 800-mg fluconazole for treatment of cryptococcal meningitis. Participants underwent intensive pharmacokinetic sampling on days 1, 5, and 14 of 1HP therapy; sampling was conducted before and 2, 4, 8, and 24 hours after dosing. Dolutegravir, rifapentine, and fluconazole plasma concentrations were measured by means of high-performance liquid chromatography mass spectrometry, and bioequivalence was assessed with standard 90% confidence intervals (CIs).

**Results:**

Fifteen participants were enrolled, of whom 9 (60%) were female. The median age (interquartile range) was 37 (27–41) years, and the median CD4 cell count was 11/μL (10–25/μL). Overall, 14 of 15 participants completed sampling on days 5 and 14. The day 14 geometric mean (GM) dolutegravir trough concentration (95% CI) was 0.61 (.32–1.16) mg/L, while the median (interquartile range) was 0.63 (0.34–0.87) mg/L, with 100% of samples above the dolutegravir protein–adjusted 90% inhibitory concentration of 0.064 mg/L, with a GM ratio of 0.76 (90% CI, .4–1.46) compared with day 1. The day 14 maximum plasma concentration and area under the concentration-time curve from 0 to 24 hours for fluconazole were reduced compared with day 1, with GM ratios of 0.73 (90% CI, .57–.94) and 0.77 (.64–.93), respectively. Of 14 participants alive at 12 weeks after 1HP initiation, 11 (78%) had an HIV viral load (VL) <200 copies/mL. No cases of cryptococcal meningitis relapse occurred during the 18-week follow-up.

**Conclusions:**

Standard once-daily 50-mg dolutegravir and 800-mg fluconazole dosing may be a safe and effective option for patients receiving 1HP tuberculosis-preventive therapy. These findings support further evaluation in larger studies.

## Teaser

Among adults treated for HIV-associated cryptococcosis, coadministration of 1-month daily isoniazid/rifapentine (1HP) modestly reduced dolutegravir trough concentrations and fluconazole exposure, yet 93% achieved virological suppression by 12 weeks, with no cryptococcal relapses during follow-up.

Tuberculosis is treatable and preventable, yet it remains the most frequent cause of AIDS-related hospital admissions and deaths worldwide [1, 2]. Despite clear recommendations and robust data showing that tuberculosis-preventive therapy (TPT) prevents tuberculosis disease and deaths [3], provision of TPT has been suboptimal, particularly for people living with advanced human immunodeficiency virus (HIV-1) disease (CD4 cell count <200/μL or World Health Organization [WHO] stage 3 or 4). Barriers to TPT implementation are multifactorial and include concerns about TPT drug toxicity, adherence, and loss to follow-up [4]. These concerns were particularly relevant with respect to the historically long duration of TPT regimens (6 or 9 months of isoniazid). Novel shorter-course TPT regimens, including 3 months of weekly isoniazid and rifapentine (3HP) and 1 month of daily rifapentine and isoniazid (1HP), may overcome some of these barriers.

Ultrashort-course 1HP TPT was endorsed by the WHO as a TPT option in 2020 [2], following the landmark BRIEF-TB trial [4]. The BRIEF-TB trial compared 1HP with 9 months of isoniazid among 3000 people living with HIV, 1HP was shown to be noninferior to 9 months of isoniazid for the primary end points of diagnosis of tuberculosis, death from tuberculosis, or death from unknown cause. In addition, the 1HP arm had significantly fewer adverse events and the highest-ever treatment completion rates reported in a TPT trial (97% in the 1HP arm).

1HP is a short, efficacious, well-tolerated, and safe TPT regimen. However, rifapentine-based TPT regimens present a potential challenge with respect to the known drug-drug interaction between rifapentine and dolutegravir, a central component of standard first-line antiretroviral therapy (ART) regimens globally. Dolutegravir is metabolized largely via uridine diphosphate glucosyltransferase 1A1, while CYP3A4 is involved to a lesser extent [5]. Rifapentine is a potent inducer of cytochrome P450 (CYP) enzymes; coadministration of rifapentine with dolutegravir is therefore expected to decrease dolutegravir concentrations, which may result in lack of HIV viral control and emergence of dolutegravir drug resistance. There are limited pharmacokinetic data to inform dosing regimens when dolutegravir is coadministered with 1HP, with clinical equipoise regarding once-daily 50-mg versus twice-daily 50-mg dolutegravir. Current WHO guidelines do not recommend dose adjustment when dolutegravir is coadministered with 1HP or 3HP [2].

The WHO guidelines were informed by several studies [6–8], including the DOLPHIN trial, a pharmacokinetic study investigating the coadministration of 3HP and dolutegravir among people living with HIV in South Africa. DOLPHIN trial participants received once-daily 50-mg dolutegravir for 8 weeks, then began once-weekly HP (rifapentine 900 mg plus isoniazid 900 mg) for 12 weeks. The DOLPHIN trial demonstrated that although coadministration with 3HP decreased dolutegravir bioavailability, which was associated with a modest decrease in dolutegravir trough levels, all trough levels except 1 were above the dolutegravir 90% inhibitory concentration. Furthermore, all HIV viral loads (VLs) measured at baseline and at 11 and 24 weeks were suppressed (defined as <40 copies/mL) in all DOLPHIN study participants [9]. ACTG A5372 found that twice-daily 50-mg dolutegravir when coadministered with 1HP had higher trough concentrations compared with once-daily dolutegravir in the absence of 1HP. No pharmacokinetic study has evaluated once-daily dolutegravir in the context of 1HP [10]. We hypothesized that since CYP450 enzyme induction takes approximately 2 weeks from treatment initiation to reach maximal effect, the shorter duration of 1HP may result in more modest decreases in dolutegravir trough levels.

In parallel, due to CYP enzyme induction, rifapentine-based 1HP may also increase the metabolism of fluconazole. It is estimated that 20% of fluconazole metabolism is via CYP3A4 [11]. Among patients receiving treatment for HIV-associated cryptococcal meningitis, a decrease in fluconazole plasma concentration during the consolidation phase of treatment due to 1HP coadministration may in theory lead to lack of cryptococcal control, and cryptococcal meningitis disease relapse. Current expert guidelines, however, do not recommend dose adjustment when fluconazole is coadministered with 1HP [12].

Here we present the results of a pharmacokinetic study in 15 adults with advanced HIV disease and cryptococcal meningitis receiving once-daily 50-mg dolutegravir-based ART and once-daily 800-mg fluconazole during 1HP therapy. We sought to determine whether 1HP coadministration leads to clinically significant reductions in dolutegravir and fluconazole exposures among adults receiving consolidation therapy for HIV-associated cryptococcal meningitis.

## METHODS

### Study Design

A single-arm, single-center, pharmacokinetic substudy nested within the IMPROVE (Integrated management of cryptococcal and opportunistic infections to improve outcomes in advanced HIV disease; ISRCTN18437550) randomized controlled trial. The IMPROVE trial was a noninferiority randomized trial that evaluated 2 different strategies for the delivery of 1HP TPT for adults receiving treatment for HIV-associated cryptococcal meningitis. The IMPROVE trial has been described elsewhere [13, 14]; in brief, consecutive hospitalized adults (aged ≥18 years) living with HIV and receiving treatment for cryptococcal meningitis (cerebrospinal fluid cryptococcal antigen positive) were screened for eligibility.

Patients were excluded if they had evidence of active tuberculosis disease. Tuberculosis screening included urine tuberculosis-lipoarabinomannan urine assay (Alere), urine Xpert MTB/Rif Ultra (Cepheid), BD Bactec Myco/F blood cultures, and chest radiography for all potential participants. Additional Xpert Ultra testing on sputum, cerebrospinal fluid, or both was conducted at the clinician's discretion, depending on the clinical syndrome and sample availability. Additional radiology, including brain imaging and abdominal ultrasonography, was also performed to evaluate for extrapulmonary tuberculosis or alternative infections at the physician’s discretion.

Patients were excluded if they had evidence of active hepatitis B infection (hepatitis B surface antigen positive), had abnormal liver function results (bilirubin >3.5 mg/dL or alanine aminotransferase >200 IU/L), had known chronic liver disease, were jaundiced, were pregnant or breastfeeding, or presented with a clinical syndrome that, in the opinion of the attending clinician, put the patient at significant risk if they were to participate in the 1HP trial. Patients were also excluded if they were taking protease inhibitors or had a known hypersensitivity to rifamycins or isoniazid.

Consenting participants underwent randomization individually and were assigned in a 1:1 ratio to either inpatient initiation of 1HP (experimental arm) or outpatient initiation of 1HP at week 6 after cryptococcal meningitis diagnosis (standard of care). Random allocation was done with a computer-generated randomization list in Stata software, version 16, with random block sizes, stratified according to site (Kampala vs Mbarara). 1HP TPT was standardized across arms, with a 28-day course of rifapentine (600 mg/d) plus isoniazid (300 mg/d) with adjunctive pyridoxine (25 mg/d). The primary end point was tuberculosis disease–free 1HP treatment completion at 18 weeks. Follow-up was through 18 weeks after cryptococcal meningitis diagnosis. The IMPROVE pharmacokinetic substudy was designed to investigate interactions between once-daily dolutegravir, 1HP, and fluconazole.

### Study Setting and Pharmacokinetic Substudy Participants

We recruited participants at Mulago National Specialised Hospital and Kiruddu National Referral Hospital in Kampala. For this pharmacokinetic substudy, we included only those randomized to outpatient initiation of 1HP who could attend the Infectious Diseases Institute pharmacokinetic unit for 3 full pharmacokinetic sampling days (see Supplementary Methods). All participants included in the pharmacokinetic substudy received dolutegravir at 50 mg once daily as part of fixed-dose combination therapy with tenofovir disoproxil fumarate–lamivudine (300/300 mg), as per Ugandan guidelines [15].

Patients were included into the IMPROVE randomized controlled trial irrespective of ART status at the time of cryptococcal meningitis diagnosis. For participants not receiving ART at at the time of cryptococcal meningitis diagnosis, ART was initiated 4–6 weeks after initiation of treatment for cryptococcal meningitis, as per WHO guidelines (to reduce the risk of central nervous system paradoxical immune reconstitution inflammatory syndrome). The sequence of initiation of ART and tuberculosis-preventive therapy was not specified in the IMPROVE protocol and was decided on a case-by-case basis, depending on participant choice. When no specific preference was given by the participant, initiating ART at week 4 (after cryptococcal meningitis diagnosis) with initiation of 1HP at week 6 was the preferred approach.

All pharmacokinetic substudy participants were established on dolutegravir-based ART and fluconazole before enrollment into the pharmacokinetic substudy. Participants were on dolutegravir therapy for ≥3 days before entry into the pharmacokinetic substudy to ensure that a dolutegravir steady state had been achieved.

At the time of 1HP initiation, participants were receiving fluconazole 800 mg once daily as continuation therapy for HIV-associated cryptococcal meningitis; participants had been receiving fluconazole for 6 weeks at the time of 1HP initiation. ART, 1HP, and fluconazole were self-administered, and adherence was measured by patient self-report.

### Pharmacokinetic Sampling

At enrollment into the pharmacokinetic substudy, participants initiated 1HP (pharmacokinetic day 1) and continued through day 28. Participants underwent intensive pharmacokinetic sampling on days 1, 5, and 14 of 1HP therapy. These time points were selected because rifapentine-mediated enzyme induction is expected to develop progressively during 1HP treatment and to approach its maximal effect within the first 2 weeks of therapy. The principal pharmacokinetic comparisons presented therefore focus on the period during which the maximal interaction was anticipated.

Intensive pharmacokinetic sampling was conducted at before dose and 2, 4, 8, 24 hours after a directly-observed dolutegravir dose. Plasma concentrations of dolutegravir, rifapentine, and fluconazole were measured at each of these time points.

On pharmacokinetic study days, participants took directly observed 1HP, dolutegravir-based ART, and fluconazole concurrently; outside of pharmacokinetic study visits, all study medications were self-administered. Participants were asked to fast for ≥3 hours before the visit, and ideally overnight. Participants who reported any missed or incorrect dosing of dolutegravir, 1HP, or fluconazole in the 3 days before a pharmacokinetic visit had that visit rescheduled. Finally, if vomiting occurred within 0.5–4 hours after the dose of dolutegravir, 1HP, or fluconazole, the pharmacokinetic visit was rescheduled. These criteria were used to ensure 3 consecutive days of adherent drug dosing before pharmacokinetic sampling.

### Pharmacokinetic Testing

Blood collected during pharmacokinetic sampling was processed within 30 minutes, and plasma was stored at −80 degrees until analysis. Plasma dolutegravir, fluconazole, and rifapentine concentrations were determined by means of reverse-phase high-performance liquid chromatography mass spectrometry using a previously published fluconazole method with modifications to accommodate dolutegravir and rifapentine [16] (see the Supplementary Methods). The assay was validated over a calibration range of 0.5–50 mg/L. The interday precision (percentage coefficient of variation) based on quality control samples and accuracy (percentage bias) were <15%. Samples on the first run that were <0.5 mg/L were run again on a separate dolutegravir assay with a limit of quantification 0.05 mg/L [17].

### Statistical Analysis

Baseline demographics and clinical characteristics were summarized as counts and percentages for categorical data or as medians (with interquartile ranges [IQRs]) and means (with ranges) for continuous data, depending on data distributions. Medians and percentiles were used to describe the pharmacokinetic data. Noncompartmental analysis was performed using Phoenix Winnonlin 8.5.2.4 software (Certara), and statistical analysis as performed using SAS 9.4 software. The pharmacokinetic parameters—maximum plasma concentration (C_max_), area under the concentration-time curve from 0 to 24 hours (AUC_0-24h_), and plasma concentration 24 hours after a dose (trough concentration) (C_24_)—for all 3 drugs were described using geometric means (GMs) and 95% confidence intervals (CIs). GM parameters on days 5 and 14 were compared with those on day 1 using GM ratios (GMRs) with 90% CIs, per Food and Drug Administration standards for bioequivalence

### Ethical and Regulatory Approvals

Approvals for the study were obtained from the Mulago Research Ethics Board (reference MHREC 2021-25), the Uganda National Council for Science and Technology (reference HS1607ES), and the London School of Hygiene and Tropical Medicine (reference 24059). Written informed consent was obtained from all participants.

## RESULTS

Fifteen participants were enrolled in the IMPROVE pharmacokinetic substudy. Their demographics are reported in Table 1. Their median age (IQR) was 37 (27–41) years, and 60% (9 of 15) were female. The median CD4 cell count (IQR) at time of cryptococcal meningitis diagnosis was 11/µL (10–25/µL). All 15 participants completed the day 1 visit, and 14 participants completed the days 5 and 14 visits.

**Table 1.** Baseline Demographic, Clinical, and Laboratory Features

Baseline Variable at Meningitis Diagnosis^a^	Participants, No. (%)^b^ (N = 15)
Female sex	9 (60)
Age, median (IQR), y	37 (27–41)
Receiving HIV ART at time of cryptococcal meningitis diagnosis	3 (20)
Duration of on HIV therapy at time of cryptococcal meningitis diagnosis, median (IQR), mo	37.5 (3.7–62.9)
Clinical presentation	
Weight, median (IQR), kg	60 (50–68)
Symptoms	
Fever	7 (46.7)
Weight loss	8 (53.3)
Night sweats	6 (40.0)
Cough	10 (66.7)
History of TPT	1 (6.7)
Earlier history of tuberculosis disease	1 (6.7)
Laboratory parameters, median (IQR)	
CD4 cell count, cells/μL	11 (10–25)
Hemoglobin, g/dL	10.9 (9.7–12.5)
Creatinine, mg/dL	0.68 (0.59–0.81)

Abbreviations: ART, antiretroviral therapy; HIV, human immunodeficiency virus; IQR, interquartile range, TPT, tuberculosis-preventive therapy.

^a^Baseline HIV viral load (VL) testing was not performed as part of the study. Among 3 participants with available HIV VLs measured at the time of HIV-associated cryptococcal meningitis diagnosis, the median VL (IQR) was 115 000 (14 800–230 000) copies/mL.

^b^Data represent no. (%) of participants unless otherwise specified.

At the time of cryptococcal meningitis diagnosis and enrollment into the IMPROVE trial, 20% of participants (3 of 15) had been receiving ART for a median duration (IQR) of 37.5 (3.7–62.9) months. At commencement of the pharmacokinetic substudy, which started at week 6 following diagnosis and initiation of antifungal treatment for cryptococcal meningitis, all 15 participants were receiving dolutegravir at 50 mg once daily as part of fixed-dose combination therapy with tenofovir disoproxil fumarate–lamivudine (300/300 mg).

### Dolutegravir Pharmacokinetics

**Figure 1.**
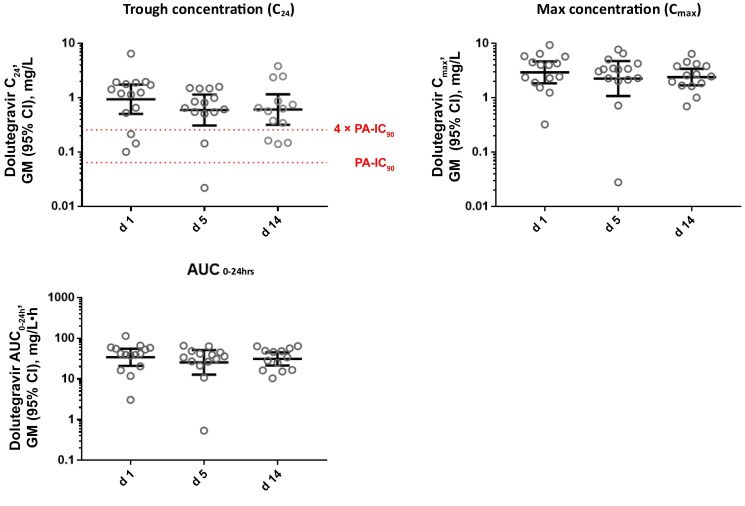
Pharmacokinetic parameters of dolutegravir plasma concentrations at days 1, 5, and 14 of 1HP (1 month of daily rifapentine and isoniazid) administration. Abbreviations: AUC_0-24h_, area under the concentration-time curve from 0 to 24 hours; C_24_, plasma concentration 24 hours after dose (trough concentration); CI, confidence interval; C_max_, maximum plasma concentration; GM, geometric mean; PA-IC_90_, protein-adjusted 90% inhibitory concentration (0.064 mg/L).

Dolutegravir, fluconazole, and rifapentine pharmacokinetic parameters are summarized in Table 2. The GM dolutegravir C_24_ after 14 days of coadministration with 1HP was 0.61 mg/L, which is 9-fold higher than the dolutegravir protein-adjusted 90% inhibitory concentration (PA-IC_90_) of 0.064 mg/L. The median (IQR) at day 14 was 0.63 (0.34–0.87) mg/L. Although 1 participant exhibited a day 5 trough concentration below the PA-IC_90_, all trough concentrations on days 1 and 14 exceeded the PA-IC_90_. In total, trough concentrations below 4 times the PA-IC_90_ were observed in 3 participants (20%) on day 1, in 2 participants (13%) on day 5, and in 3 participants (21%) on day 14. After 5 days of coadministration with 1HP, the dolutegravir C_24_ GMR between days 5 and 1 was 0.64 (90% CI, .43–.95). By day 14, this effect was less pronounced, with the C_24_ GMR 0.76 (90% CI, .40–1.46) (Figure 1 and Supplementary Figure 1).

**Table 2.** Dolutegravir and Fluconazole Pharmacokinetic Parameters Across 14 Days of 1HP Coadministration

Parameter	d 1 of 1HP		d 5 of 1HP		d 5 vs d 1	d 14 of 1HP		d 14 vs d 1 GMR (90% CI)
	Participants, No.	GM (95% CI)	Participants, No.	GM (95% CI)	GMR (90% CI)	Participants, No.	GM (95% CI)	
Dolutegravir								
C_max_, mg/L	15	2.93 (1.85–4.65)	15	2.26 (1.08–4.75)	0.85 (.63–1.15)	14	2.41 (1.70–3.43)	0.81 (.49–1.34)
AUC_0-24h_, mg/L ⋅ h	15	34.17 (21.08–55.57)	14	25.65 (12.81–51.36)	0.76 (.57–1.00)	13	31.23 (21.52–45.33)	1.01 (.61–1.69)
C_24_, mg/L	15	0.94 (.50–1.74)	14	0.59 (.31–1.15)	0.64 (.43–.95)	13	0.61 (.32–1.16)	0.76 (.40–1.46)
Fluconazole								
C_max_, mg/L	15	66.8 (49.8–89.5)	15	63.0 (43.8–90.7)	0.94 (.42–1.24)	15	48.7 (32.2–73.8)	0.73 (.57–.94)
AUC_0-24h_, mg/L ⋅ h	15	1072 (749.7–1532.9)	14	993 (661.1–1491.6)	0.89 (.7–1.12)	13	840.4 (519.2–1360.3)	0.77 (.64–.93)
C_24_, mg/L	15	32.1 (14.7–70.2)	14	24.5 (10.2–59.3)	0.73 (.30–1.79)	13	34.2 (20.3–57.7)	1.06 (.63–1.78)

Abbreviation: 1HP, 1 month of daily rifapentine and isoniazid; AUC_0-24h_, area under the concentration-time curve from 0 to 24 hours; C_24_, plasma concentration 24 hours after dose (trough concentration); CI, confidence interval; C_max_, maximum plasma concentration; GM, geometric mean; GMR, GM ratio.

The dolutegravir AUC_0-24h_ was likewise reduced on day 5 (GMR, 0.76 [90% CI, .57–1.00]); whereas dolutegravir AUC_0-24h_ values on day 14 were comparable to those on day 1. The dolutegravir C_max_ remained largely unchanged throughout the study period.

### Fluconazole and Rifapentine Pharmacokinetics

The GM fluconazole C_24_ remained comparable at both days 5 and day 14 compared with day 1 (Table 2). After 14 days of coadministration with 1HP, the GM fluconazole C_24_ was 34.2 mg/L (90% CI, 20.3–57.7 mg/L) compared with 32.1 mg/L (14.7–70.2 mg/L) on day 1 (GMR, 1.06 [.63–1.78]).

In contrast, reduction in fluconazole C_max_ were observed with 1HP coadministration. On day 14 of coadministration with 1HP, fluconazole C_max_ was reduced relative to baseline (GMR, 0.73 [90% CI, .57–.94]). Similarly, by day 14, the fluconazole AUC_0-24h_ was reduced (GMR, 0.77 [90% CI, .64–.93]). Assuming a population minimum inhibitory concentration (MIC) 50 of 4 µg/mL, the probability of achieving an AUC/MIC ratio >100 decreased from 87% at day 1 to 76% by day 14 [18, 19]. As expected, evidence of rifapentine autoinduction over time was observed (Supplementary Table 1 and Supplementary Figure 1).

### HIV Virological Control and Cryptococcal Meningitis Treatment Outcomes

The 18-week mortality rate (18 weeks after cryptococcal meningitis diagnosis) within our cohort was 7% (1 of 15). Of the 14 participants alive 12 weeks after 1HP initiation, 11 (78%) had an HIV VL <200 copies/mL, and 8 (57%) had an HIV VL <50 copies/mL. Two participants with an HIV VL ≥200 copies/mL at week 12 were new ART initiators and had started dolutegravir, tenofovir disoproxil fumarate, and lamivudine at week 4 after cryptococcal meningitis diagnosis. Of the 3 participants (21%) with an HIV VL ≥200 copies/mL at week 12, 2 participants subsequently had an HIV VL <200 copies/mL by week 28 or week 32, and the remaining participant self-discontinued ART. Of the 3 participants with VLs ≥200 copies/mL, 2 had a dolutegravir C_24_ <0.32 mg/L on days 1 and 5 of 1HP but not day 14, suggesting their low dolutegravir exposures are more likely due to poor adherence than to 1HP. No cases of cryptococcal meningitis relapse occurred during the 18-week follow-up.

## DISCUSSION

In this pharmacokinetic study of 15 adults with advanced HIV disease receiving treatment for cryptococcal meningitis, we found that coadministration of 1HP led to modest reductions in dolutegravir trough concentrations and decreased C_max_ and AUC_0-24h_ for fluconazole by day 14. Despite these expected decreases in exposure, the day 14 GM dolutegravir C_24_ remained 9-fold above the PA-IC_90_, and 93% of participants achieved virological suppression within 32 weeks after completing 1HP. In addition, consistent with the majority of participants maintaining an AUC/MIC ratio >100 [18, 19], despite the modest decrease in fluconazole exposures, no cases of cryptococcal relapse were observed. Together, these pharmacokinetic, virological, and clinical data support the safety and effectiveness of once-daily dolutegravir 50-mg and fluconazole 800-mg dosing (for consolidative cryptococcal meningitis treatment) during 1HP administration.

Our data agree with the published literature [6, 8]. In a study of 13 adults in Thailand receiving once-daily 50-mg dolutegravir–based ART, 1HP was shown to reduce the dolutegravir minimum concentration by 24.9% on the final day of 1HP, compared with day 0 prior to 1HP; however, HIV viral suppression was maintained in all but 1 participant at 1HP completion [6]. In a second multicenter retrospective cohort study conducted in Taiwan, in which study participants received once-daily 50-mg dolutegravir–based ART and 1HP (n = 46) or 3HP (n = 214), 100% and 95.8% of 1HP and 3HP participants, respectively, had an HIV VL <200 copies/mL within 12 months of TPT completion; however, pharmacokinetic data were not presented as part of this study [8]. Importantly, reduced drug clearance in Asian populations has been reported, likely due to the higher prevalence of the decreased-function allele UGT1A1*6. While this allele is not frequent in individuals of sub-Saharan African descent, the *28 allele is detected at a relatively high frequency (40% prevalence), and it may also be associated with decreased function and slower drug clearance. Our study did not include genotyping, but these data highlight the importance of including African populations in future drug-drug interaction studies [20, 21]. Taken together with our data, these studies indicate that although rifapentine-based 1HP leads to modest reductions in dolutegravir trough concentrations, its clinical impact appears limited, as the vast majority of study participants maintained virological suppression throughout 1HP. Collectively, the available evidence suggests that once-daily 50-mg dolutegravir dosing is a potentially safe and effective option for patients receiving 1HP.

While the prevalence of HIV virological suppression within our study population was very high, 93% at 12 weeks after 1HP completion, it was lower than among study participants in the DOLPHIN study, 100% of whom achieved HIV virological suppression. The reasons for this difference are likely multifactorial, including suboptimal ART adherence among our study population. Given that 20% of participants had dolutegravir trough concentrations below 4 times the PA-IC_90_ at baseline, this indicates suboptimal ART adherence within this population of adults with advanced HIV disease receiving concurrent treatment for cryptococcal meningitis. The fact that this proportion had reduced to 13% by day 5 suggests that dolutegravir adherence may have improved during the course of the study, thereby limiting our power to detect relative reductions in dolutegravir concentrations over time. However, it is also possible that the dolutegravir plasma concentrations required to maintain virological suppression are lower than those needed to achieve initial virological suppression in individuals with uncontrolled HIV replication.

There is an urgent need to increase the reach of TPT as an intervention for persons living with advanced HIV disease. 1HP may have advantages in the context of advanced HIV disease with respect to rapid sterilization of latent tuberculosis infection, shorter overall duration, and reduced pill burden [7]. Concern around drug-drug interactions with rifapentine, however, has limited the clinical use of 1HP. Although, due to a paucity of evidence, current WHO guidelines do not recommend dolutegravir dose adjustment with 1HP, twice-daily dolutegravir dosing remains common, with programs often providing an additional evening dose of stand-alone dolutegravir for the duration of rifapentine-containing TPT, where available. This approach, while safe and effective [22], adds to the cost and pill burden and creates unnecessary logistical and financial challenges for patients and health systems. The pharmacokinetic data presented, along with the high rates of HIV virological suppression achieved in this cohort, highlight the potential for simplified once-daily dolutegravir dosing with 1HP.

Our study has several limitations. First, the relatively small sample size (n = 15) resulted in wide CIs around the pharmacokinetic estimates, limiting the precision of our findings and warranting cautious interpretation. Second, participants were recruited from a single study site in Uganda, which may limit the generalizability of the results to other populations and clinical settings. Furthermore and importantly, all participants in our study were also receiving fluconazole for consolidation therapy for HIV-associated cryptococcal meningitis. Given that rifapentine is an inducer of CYP enzymes and fluconazole is an inhibitor of certain CYP enzymes, we acknowledge that in our study population fluconazole coadministration may have partially offset some CYP-mediated effects of rifapentine on dolutegravir plasma concentrations. Third, the limit of detection for our dolutegravir assay was 0.5 mg/L, which may have affected the precision of measurements at very low concentrations. For this reason, we performed a rerun using a separate dolutegravir assay with a limit of quantification of 0.05 mg/L. A fourth limitation is that, while we compared the GM dolutegravir C_24_ at day 14 with the dolutegravir PA-IC_90_, it is important to recognize that a definitive plasma concentration threshold required for sustained virological suppression—or, conversely, a concentration below which virological failure is likely—has not been clearly established. As a result, interpreting dolutegravir pharmacokinetic data in terms of clinical impact remains problematic. Given these limitations, further evaluation of once-daily dolutegravir with 1HP in larger, diverse populations with accompanying HIV resistance testing is needed to validate our findings before broadly rolling out a once-daily dosing strategy for dolutegravir with 1HP.

In conclusion, our findings, together with emerging evidence on the concomitant use of rifapentine and dolutegravir, suggest that despite the anticipated reductions in drug exposure, once-daily 50-mg dolutegravir and 800-mg fluconazole may represent a safe and effective regimen during 1HP in adults with advanced HIV disease receiving treatment for cryptococcal meningitis.

## Supplementary Material

ofag415_Supplementary_Data

## Supplementary Data

Supplementary materials are available at *Open Forum Infectious Diseases* online. Consisting of data provided by the authors to benefit the reader, the posted materials are not copyedited and are the sole responsibility of the authors, so questions or comments should be addressed to the corresponding author.
