# Supplementary material for: Once-Daily Dolutegravir–Based Antiretroviral Therapy Among Adults With Advanced HIV Disease Receiving 1 Month of Daily Rifapentine and Isoniazid for Tuberculosis-Preventive Therapy and Fluconazole for Treatment of Cryptococcal Meningitis: Results of a Pharmacokinetic Study
Source: Open Forum Infect Dis. 2026 Jul 8;13(8):ofag415. doi: 10.1093/ofid/ofag415 (PMC13469692; doi:10.1093/ofid/ofag415)
Supplement: Supplementary file 1 [file ofag415_supplementary_data.docx]

**Supplementary materials:**

**Supplementary methods:**

**Timeline graphic of the IMPROVE randomised controlled trial**

Footnotes: 1HP = One month of rifapentine and isoniazid; TPT = TB Preventative Therapy; ART = anti-retroviral therapy

Following diagnosis of HIV-associated cryptococcal meningitis and initiation of anti-fungal therapy at day 0, participants were screened for eligibility into the IMPROVE trial. Eligible participants were randomised to inpatient or outpatient initiation of 1HP at the point of hospital discharge. Participants randomised to inpatient 1HP arm, initiated 1HP under direct observation whilst hospitalised (thereafter, 1HP was dispensed as take-home medication and self-administered in the community between study week 2-4). Participants randomised to outpatient 1HP arm, initiated 1HP under direct observation in the outpatient clinic at week-6. For this pharmacokinetic sub-study, we only included those randomised to outpatient initiation of 1HP. For participants not receiving ART at time of cryptococcal meningitis diagnosis, ART was initiated between 4-6 weeks after initiation of treatment for cryptococcal meningitis. Pharmacokinetic sampling took place between week 6-8. HIV viral load testing was conducted at week-18 for IMPROVE study participants. The HIV viral load data collected for study participants at the week-32 time point was collected as part of the observational parent study in which the IMPROVE trial was nested within.

**Supplementary methods:**

The three drugs with their respective deuterated internal standards (DTG-d4, FLU-d4 and RPT-d8) were extracted from plasma samples using protein precipitation with acetonitrile. Chromatography was performed on a Waters Atlantis 150x3mm, particle size 3µm column using a mobile phase comprised of eluents A (composed of 0.1% formic acid in water), and B (composed of 0.1% formic acid in acetonitrile). Initial conditions consisted of 70% eluent A for the first 0.5 minutes, increasing in organic content to 95% B in 1.5 min, kept at 95% of B till 3 minutes and equilibrated back to the initial conditions over a total run time of 6.5 min. The flow rate was 400 µl/min. The 3-D ion trap mass spectrometer (LCQ-FLEET; Thermo Scientific, Waltham Massachusetts, USA) was operated in positive ionization mode. Analyte detection and quantification was performed using selective reaction monitoring (SRM) using transitions: dolutegravir, 420.2→277; fluconazole, 307.2→237.8; rifapentine, 877.6→845.5; dolutegravir-d4, 424.2→279.1; fluconazole-d4, 311.2→241.8 and rifapentine-d8, 885.6→852.8. The collision energy was the same for the analytes and their respective internal standards; 25, 21 and 17 for dolutegravir, fluconazole and rifapentine respectively.**Supplementary table 1: Rifapentine pharmacokinetic parameters across 14 days of coadministration**

|  | **Day 1 of 1HP** | | **Day 5 of 1HP** | | **Day 14 of 1HP** | |
| --- | --- | --- | --- | --- | --- | --- |
|  | n | Geometric mean  (95% CI) | n | Geometric mean  (95% CI) | n | Geometric mean  (95% CI) |
| Rifapentine  C_max,_ mg/L | 15 | 12.8  (9.2-17.8) | 15 | 22.6  (17.0-29.9) | 14 | 16.9  (11.9-24.0) |
| Rifapentine AUC_0-24h,_ mg/L*hr | 15 | 196.2  (141.7-271.6) | 14 | 349.2  (224.8-542.4) | 12 | 257.3  (117-373.9) |
| Rifapentine C_24,_ mg/L | 15 | 6.5  (4.3-9.8) | 14 | 10.7  (5.1-22.6) | 12 | 8.8  (5.9-12.2) |

**Supplementary figure 1: Plasma concentration–time profiles of dolutegravir, fluconazole, and rifapentine on days 1, 5, and 14 of 1HP therapy**

Footnotes:

1HP = One month of rifapentine and isoniazid.
